# miR-122 Targets Pyruvate Kinase M2 and Affects Metabolism of Hepatocellular Carcinoma

**DOI:** 10.1371/journal.pone.0086872

**Published:** 2014-01-23

**Authors:** Angela M. Liu, Zhi Xu, Felix H. Shek, Kwong-Fai Wong, Nikki P. Lee, Ronnie T. Poon, Jinfei Chen, John M. Luk

**Affiliations:** 1 Department of Pharmacology, National University of Singapore, Singapore; 2 Department of Surgery, National University Health System, Singapore; 3 Department of Oncology, Nanjing First Hospital, Nanjing Medical University, Nanjing, China; 4 Cancer Science Institute, National University of Singapore, Singapore; 5 Department of Surgery, The University of Hong Kong, Queen Mary Hospital, Hong Kong; H.Lee Moffitt Cancer Center & Research Institute, United States of America

## Abstract

In contrast to normal differentiated cells that depend on mitochondrial oxidative phosphorylation for energy production, cancer cells have evolved to utilize aerobic glycolysis (Warburg’s effect), with benefit of providing intermediates for biomass production. MicroRNA-122 (miR-122) is highly expressed in normal liver tissue regulating a wide variety of biological processes including cellular metabolism, but is reduced in hepatocellular carcinoma (HCC). Overexpression of miR-122 was shown to inhibit cancer cell proliferation, metastasis, and increase chemosensitivity, but its functions in cancer metabolism remains unknown. The present study aims to identify the miR-122 targeted genes and to investigate the associated regulatory mechanisms in HCC metabolism. We found the ectopic overexpression of miR-122 affected metabolic activities of HCC cells, evidenced by the reduced lactate production and increased oxygen consumption. Integrated gene expression analysis in a cohort of 94 HCC tissues revealed miR-122 level tightly associated with a battery of glycolytic genes, in which *pyruvate kinase* (*PK*) gene showed the strongest anti-correlation coefficient (*Pearson r* = −0.6938, *p* = <0.0001). In addition, reduced *PK* level was significantly associated with poor clinical outcomes of HCC patients. We found isoform M2 (PKM2) is the dominant form highly expressed in HCC and is a direct target of miR-122, as overexpression of miR-122 reduced both the mRNA and protein levels of *PKM2*, whereas PKM2 re-expression abrogated the miR-122-mediated glycolytic activities. The present study demonstrated the regulatory role of miR-122 on *PKM2* in HCC, having an implication of therapeutic intervention targeting cancer metabolic pathways.

## Introduction

Hepatocellular carcinoma (HCC) is a common malignant tumor. In 2008, there were over 700,000 new incidences diagnosed worldwide [1]. HCC patients usually have poor clinical outcome – only 5–9% of them survive five years or more. Surgical resection, liver transplantation, and radiofrequency ablation may provide cure for some early staged patients, but most patients are unfortunately diagnosed at advanced stage given the asymptomatic nature of HCC. Moreover, HCC is highly resistant to chemoregimens, thus many of the patients die from disease recurrence. New therapeutic is in need. In recent years, miRNA has emerged as an important class of gene regulator in HCC development, and the investigation of its relevant regulatory mechanisms might provide new targets for the therapeutic intervention.

As regarded as a liver-specific non-coding RNA, miR-122 is highly expressed in normal liver tissue, but its expression level progressively reduced in cirrhotic and HCC tissues [2]. Recently, a mouse model with germline deletion of *miR-122a* showed promoting epithelial-mesenchymal transition (EMT) and spontaneous HCC formation [3]. In xenograft mouse models, miR-122 was demonstrated to affect HCC intrahepatic metastasis by angiogenesis suppression [4]. Restoration of miR-122 in HCC cells could suppress tumorigenic phenotypes, such as cell proliferation, migration, invasion, and anchorage-independent growth [5]. Recently, molecular profiling of human HCC tumors at gene and protein levels have shed light on the relationship between miR-122 and liver metabolism [2], [6]. These studies have shown that the networks of genes/proteins that correlated with miR-122 expression were enriched for functions associated with metabolic processes. Notably, mice knockout of miR-122 developed HCC with extensive lipid accumulation and reduced glycogen storage [3], implying the tumor suppressive role of miR-122 via modulating cancer metabolism.

Alteration of glycolytic metabolism is a common feature of cancer cells. Contrary to normal differentiated cells that use mitochondrial oxidative phosphorylation as a main source for energy production, cancer cell is addictively dependent on glycolysis – a phenomenon firstly reported by Otto Warburg who observed that tumor cells showed high glycolytic rate with production of lactate even in an oxygen-rich condition [7]. The phenomenon was coined as Warburg effect or aerobic glycolysis. This shift in metabolism is believed to provide metabolic needs for the rapid proliferating cancer cells to grow, rather than energy production [8]. The understanding of the control of this metabolic shift is pivotal to identify potential targets for cancer therapeutics, and the present study is to identify and characterize the miR-122-targeted metabolic genes with an attempt to evaluate the potential of reversing aerobic glycolysis in HCC.

## Materials and Methods

### Clinical specimens and cell culture

Human HCC clinical samples were collected from patients who had hepatectomy for treatment of HCC at Queen Mary Hospital, Pokfulam, Hong Kong. Clinicopathologic features are summarized in Table 1 and elsewhere [2]. Liver cancer cell lines (HepG2, Hep3B, Huh-7, H2P, H2M, MHCC97L, and MHCC97H) were obtained as previously described [9], [10]. Cells were grown in Dulbecco’s modified Eagle’s medium (DMEM) (Invitrogen, Carlsbad, CA) supplemented with 10% fetal bovine serum (Invitrogen) at 37°C in a 5% CO_2_ incubator.

**Table 1:** Clinical correlation between *PK* transcriptional expression and clinicopathological parameters of HCC patients (n = 217).

Variables	Frequency (%)	*PK* expression		*P* values
		< median	≥ median	
Sex				
Male	171 (78.8)	89	82	0.302
Female	46 (21.2)	20	26	
Age				
< 55	111 (51.2)	57	54	0.735
≥ 55	106 (48.8)	52	54	
Tumor size, cm				
< 5	86 (39.6)	46	40	0.437
≥ 5	131 (60.4)	63	68	
Alpha fetoprotein, ng/mL				
< 100	108 (49.8)	65	43	***0.004***
≥ 100	109 (50.5)	44	65	
HBsAg				
Negative	30 (13.8)	17	13	0.448
Positive	187 (86.2)	92	95	
1Histological differentiation				
Well	37(19.4)	25	12	***0.033***
Moderate/Poor	154 (80.6)	74	80	
TNM stage				
Early (I, II)	98 (45.2)	60	38	***0.003***
Late (III, IV)	119 (54.8)	49	70	
Cirrhosis				
Negative	90 (41.5)	47	43	0.621
Positive	127 (58.5)	62	65	
Venous infiltration				
Absent	107 (49.5)	65	42	***0.002***
Present	109 (50.5)	43	66	
Recurrence				
Absent	90 (41.5)	49	41	0.296
Present	127 (58.5)	60	67	

1 Analysis on 191 patients.

### RNA isolation, microarray analysis, and real-time quantitative PCR

Total RNA was extracted from cells and human HCC tumor samples using Trizol (Invitrogen), followed by reverse transcription [2]. MiRNA profiling was performed as previously described [2]. Real-time quantitative PCR (qPCR) detection of genes in cell lines was performed using 2X Power SYBR Green Master Mix (Applied Biosystems, Foster City, CA) according to manufacturer’s instructions. Detection of miR-122 in cell lines was performed using TaqMan microRNA assays according to manufacturer’s instructions (Part number: 4427975, Applied Biosystems). Transcriptional expressions of PKM1, PKM2 and miR-122 were referenced to 18S housekeeping gene. Experiments were conducted in duplicates in three independent assays.

### Western blotting

Western blot analysis was performed as described previously [11]. The membranes were blotted with antibodies of PKM2 (Cell signaling Inc., Beverly, MA), or β-actin (Sigma-Aldrich, Saint Louis, MO).

### Immunohistochemistry

Paraffin-embedded tissue blocks were sectioned for immunohistochemical staining. After antigen retrieval and peroxidase blocking, the sections were incubated with human PKM2 antibody (Cell signaling Inc.) in 1:600. The rest of the steps were performed as described [12].

### Construction of expression vectors

The miR-122 expression vector (pc-miR-122) was constructed as previously described [11]. The PKM2 expression vector was constructed by PCR amplification of the entire coding region of *PKM2*, but lacked the 5′- and 3′-UTR region. The amplified fragment was cloned into pcDNA3.1 using *NotI* and *EcoRI*. The expression vector was subsequently verified by DNA sequencing, and was called pc-PKM2 hereafter. The miR-122 inhibitor (hsa-miR-122-5p, Catalog # 4464084 ), siRNA targeting PKM2 (siPKM2, Catalog # AM51331) and non-targeting siRNA (Silencer Negative control #1 siRNA, siCtrl) were purchased from Invitorgen.

### Lactate assay

Cells were seeded onto a 6-well plate. For each well, 4 µg of pcDNA3.1 or pc-miR-122 was transfected using 5 µl of Lipofectamine 2000 (Invitrogen) according to manufacturer’s instruction. Medium was changed 24-hour post-transfection. Lactate assay (BioVision, Mountain View, CA) was then performed to quantify the cellular lactate level at 48-hour post-transfection, according to manufacturer’s instructions. The plate was read at OD570, and the amount of lactate was expressed as nmol/1×10^6^ cells.

### Cell viability assay

Cells were seeded onto 6-well plate, and transfection was performed on the next day as mentioned above. At 24-hour post-transfection, cells were trypsinized and seeded onto 96-well plates at a density of 5,000 cells/well. At 48-hour post-transfection, the plates were subjected to different culturing conditions: normoxia, hypoxic chamber with 1% oxygen, addition of 100 uM desferrioxamine (DFO), or addition of 200 ng/ml Oligomycin (Sigma-Alrich). At 72-hr post-transfection, a MTT assay was performed to measure cell viability.

### Oxygen consumption assay

We used MitoXpress-Xtras HS (Luxcel Biosciences, Cork, Ireland) to quantify the oxygen consumption of the transfected HepG2. The assay was based on the phosphorescent oxygen sensitive probe that is quenched by O_2_ at the excited state. At 48-hr post-transfection, MitoXpress probe was added to each well of the 96-well plate and the signals were measured according to the manufacturer’s instructions. The fluorescence-time profiles of different treatments were linearized using the following coordinate scale [Y: I(t_0_)/(I(t) –I(t_0_)); X: 1/t (min^−1^)], in which I(t_0_) represents fluorescence intensity at the start, and I(t) represents the intensity at time t of monitoring. Linear regression was applied to each transformed profile and the slope represents the oxygen consumption rate.

### Statistical analyses

A Student *t*-test was performed to compare two groups, and *p* ≤ 0.05 was considered significant. Kaplan-Meier plots and log-rank tests were used for survival analysis.

### Ethics statement

The study protocol was approved by the Internal Review Board of the Joint Ethics Committee of The University of Hong Kong and the Queen Mary Hospital (Pokfulam, Hong Kong), and all HCC patients have given written informed consents on the use of clinical specimens for medical research.

## Results

### miR-122-mediated reversal of aerobic glycolysis in HCC cells

**Figure 1:**
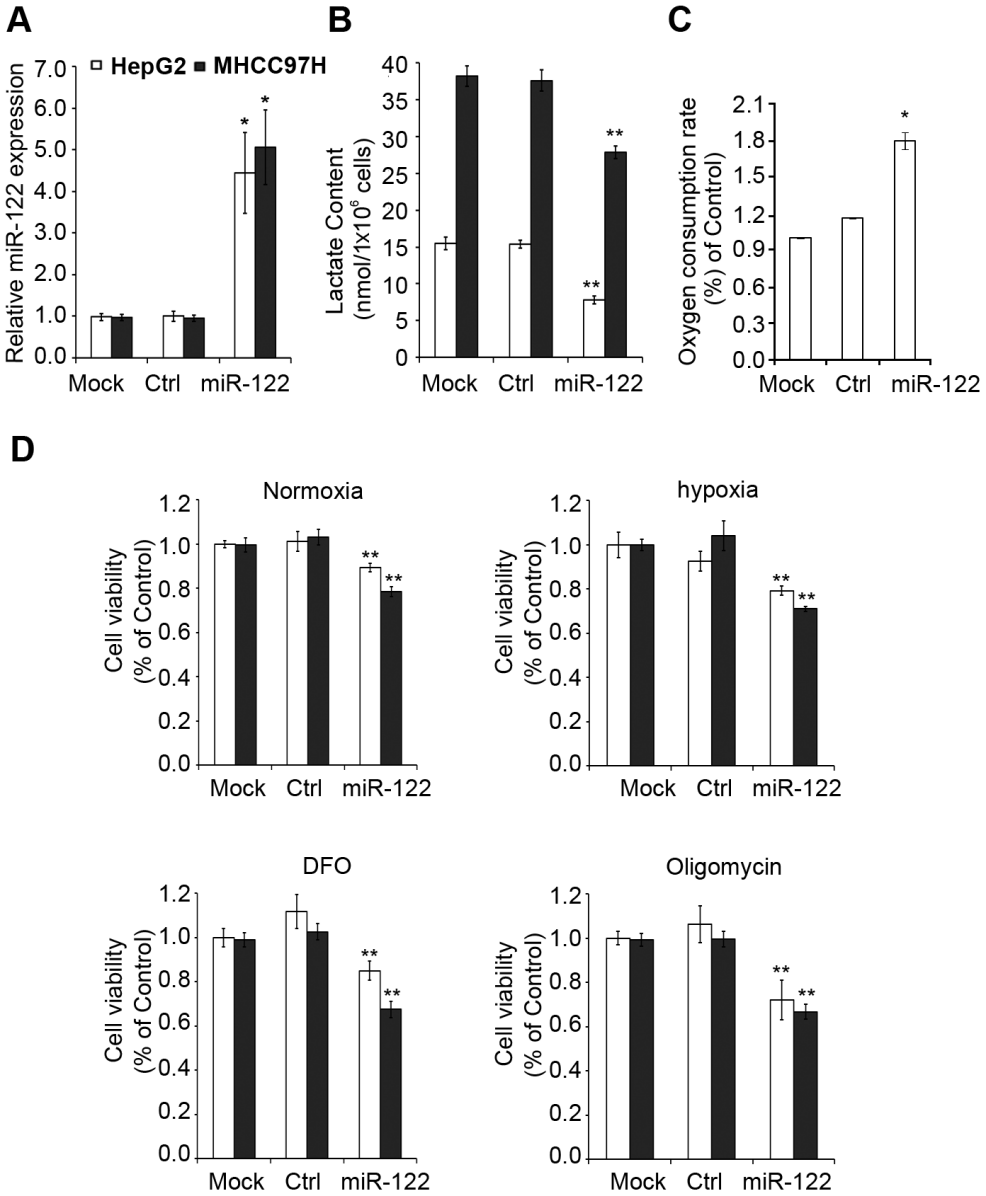
miR-122 expression resulted in reduced aerobic glycolysis of HCC cells. (**A**) Expression of miR-122 detected by qPCR 48-hour post-transfection in HepG2 or MHCC97H cells. Mock: transfection reagent only; Ctrl: transfection with empty-vector; miR-122: transfection with pc-miR-122. (**B**) Lactate contents of miR-122 or Ctrl treated cells. (**C**) Oxygen consumption rate of miR-122 or Ctrl treated cells. (**D**) Cell viability of miR-122 or Ctrl treated cells under different culturing conditions: normoxia (21% oxygen), 100 uM desferrioxamine (DFO), hypoxia (1% oxygen), or 100 ng/ml of oligomycin. * *p*<0.05, ** *p*<0.01, compared to respective Ctrl. Error bars represent S.E.M.

To evaluate the potential of miR-122 reversing aerobic glycolysis, we conducted lactate production and oxygen consumption assays in HepG2 and MHCC97H cells (with low endogenous miR-122 level) transfected with miR-122 construct or vector control. The miR-122 transfectants gave, as determined by real-time qPCR, a boost of a 4–5 folds increase of miR-122 level as compared to the vector (Fig. 1A), whereas the lactate production was reduced (Fig. 1B) and oxygen consumption in HepG2 cells was increased (Fig. 1C), indicating a metabolic shift from glycolysis to oxidative phosphorylation.

We next investigated the tumor suppressive phenotype (cell viability assay) in the miR-122 transfectants with respect to oxidative phosphorylation under different culturing conditions. We found the miR-122 transfectants had reduced cell viability under normoxia (Fig 1D), confirming observation from others [5], and interestingly, further reduction in cell viability of miR-122 transfectants was observed under hypoxia (1% oxygen), by addition of DFO or oligomycin (Fig. 1D). Oligomycin is a specific inhibitor of mitochondrial ATP synthase, and DFO is an iron chelator that is commonly used to induce the effects of hypoxia. Compared to the normoxia environment, cells grown under the above-mentioned three conditions had compromised oxidative phosphorylation. The further reduction of cell viability in these conditions suggests miR-122 cells were more dependent on oxidative phosphorylation to support cell growth. Together, the present findings supported the role of miR-122 on reversing the aerobic glycolysis to normal physiologic oxidative phosphorylation in HCC cells.

### Pyruvate kinase (PK) is anti-correlated with miR-122 expression and associated with clinical outcome

**Figure 2:**
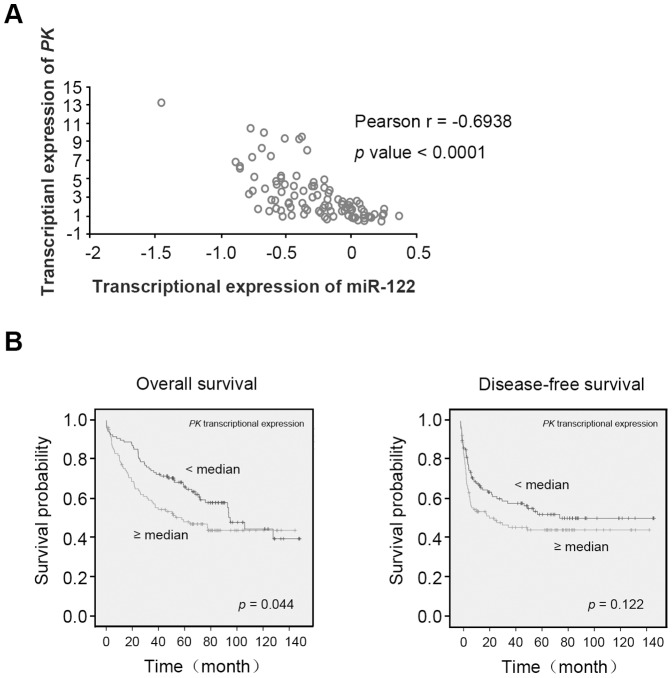
*PK* is anti-correlated with miR-122 expression in HCC tumors and correlates with outcome of HCC patients. (**A**) Transcriptional expression correlation of *PK* and miR-122 in 94 human HCC tumor samples. (**B**) Kaplan-Meier overall survival (left panel) and disease-free survival (right panel) analyses of 217 HCC patients, based on *PK* transcript level. Patients were divided into two groups: 1) higher than the median PK expression level, 2) lower than the median PK expression level.

Since miR-122 reduced aerobic glycolysis of HCC cells, we asked which of the genes are involved in this metabolic alteration. To search for such targets, we analyzed the expression profiles of glycolytic genes that are anti-correlated with the miR-122 level in a cohort of 94 HCC samples (GEO accession number GSE22058). The findings revealed the following target genes with predicted miR-122 binding site at the 3’UTR region: *PK, ALDOA, GNPDA1, PFKFB2*, and *AKR1B10*, in which the *PK* gene has the highest anti-correlation coefficient with miR-122 (*Pearson r* = –0.6938, *p* = <0.0001) (Fig. 2A). Next we tested whether these genes were relevant to the clinical outcome of HCC patients. Among these anti-correlated glycolytic genes, the *PK* transcript level was significantly associated with the overall survival time of HCC patients (Fig. 2B), as well as the serum alpha-fetoprotein level, histological differentiation of tumor tissue, and TNM stage (Table 1).

### PKM2 is highly expressed in HCC cell lines and clinical samples

**Figure 3:**
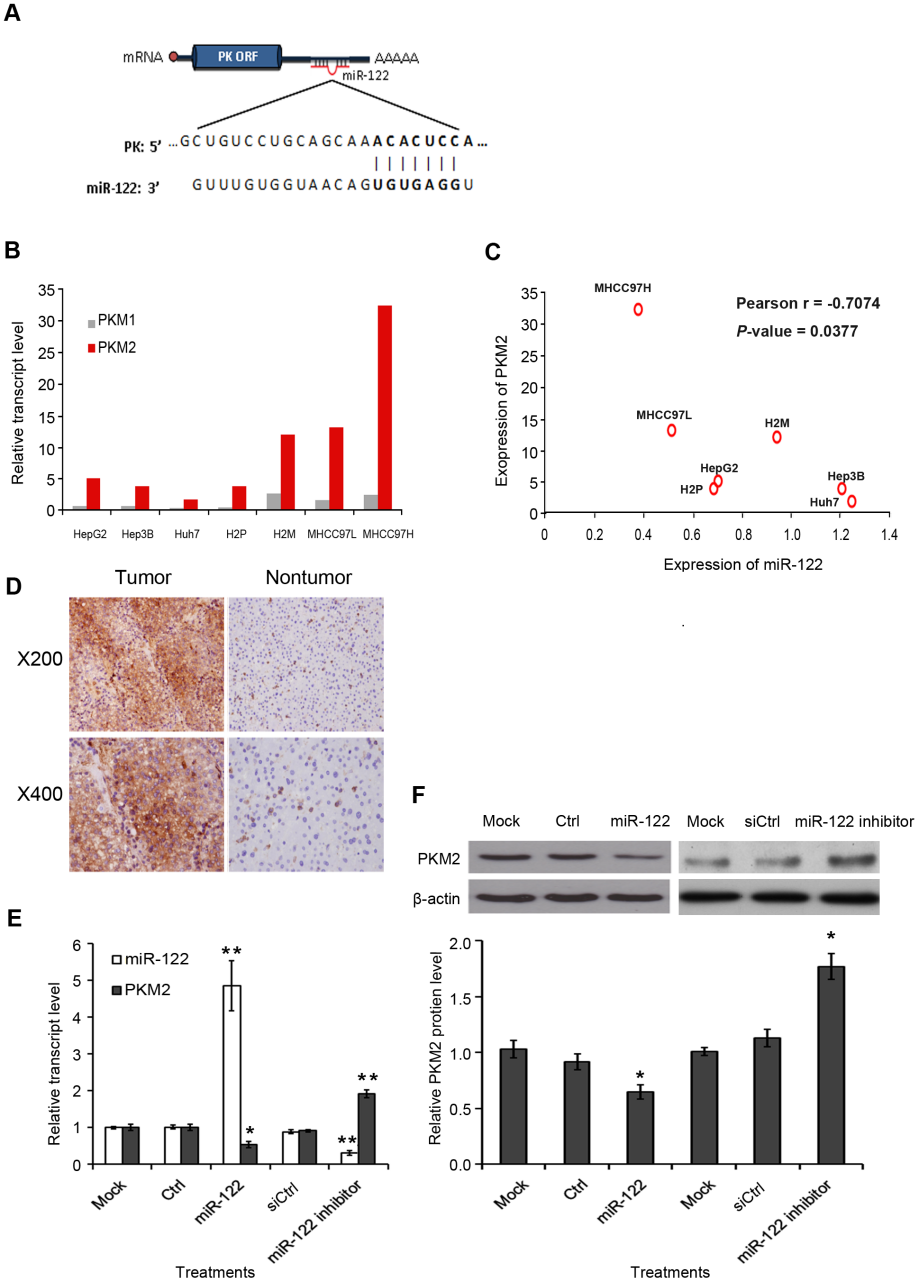
PKM2 is a direct target of miR-122. **(A)** Predicted binding interaction between 3’UTR of PK and miR-122. (**B**) Expressions of *PKM1* and *PKM2* in HCC cell lines, as detected by qPCR method. (**C**) Expression correlation of *PKM2* and miR-122 in a panel of HCC cell lines. (**D**) Immunohistochemical staining of anti-PKM2 antibody in paired tumor and adjacent nontumor tissue. A total of 12 tumor/nontumor tissue pairs were tested, and representative photomicrographs are shown. (**E**) Expression level of *PKM*2 mRNA and miR-122 levels in Ctrl, miR-122, siCtrl or miR-122 inhibitor treated HepG2 cells. Mock: transfection reagent only; Ctrl: transfection with empty-vector; miR-122: transfection with pc-miR-122; siCtrl: transfection with non-targeting siRNA; miR-122 inhibitor: transfection with miR-122 inhibitor. Data were derived from three independent experiments. (**F**) Immunoblots (upper panel) of protein level of PKM2 after transfection of miR-122 or miR-122 inhibitor in HepG2 cells. The intensities are quantified and represented in the graph in lower panel. Data were derived from two independent experiments. *p<0.05, **p<0.01, Error bars represent S.E.M.

The *PK* gene has two splice variants in humans: M1 and M2. Both isoforms entail the same 3’UTR sequence, and therefore the same miR-122 binding site. The miRNA target prediction tool, TargetScan 5.1, showed that miR-122 is the only miRNA species that binds to the 3’UTR of *PK* gene (Fig. 3A). Interestingly, the M2 variant (PKM2) was the dominant form in HCC cell lines whereas the expression of M1 variant (PKM1) was minimal (Fig. 3B), suggesting PKM2 is the tumorigenic form and highly expressed in tumors in agreement with a previous study [13]. Among the HCC cell lines tested, PKM2 generally exhibited higher expression levels in the metastatic cell lines than the primary HCC cell lines. In addition, the negative correlation in expression between *PKM2* and miR-122 was recapitulated in a panel of HCC cell lines, with the *Pearson r* value of –0.7074 (*p* = 0.0377) (Fig. 3C), similar to that observed in the clinical samples. Further confirmation of PKM2 protein expression in HCC tumors was conducted with PKM2 specific monoclonal antibody by immunohistochemical staining. Among the 12 pairs of tumor and non-tumor tissues tested, 5 tumor samples were scored at strong (++) expression, 4 samples at moderate (+), and 3 samples at minimal/undetectable (–) levels for PKM2 (Fig. 3D, Table 2). The following study focused on the oncogenic functions of *PKM2* variant in HCC.

**Table 2:** PKM2 expression in 12 HCC tumor tissue.

PKM2	Stage I-II	Stage III-IV	Total
–	2	1	3
+/++	4	5	9
Total	6	6	12

### miR-122 targets PKM2 and regulates its expression

To validate *PKM2* as a direct target of miR-122, we transfected miR-122 expression vector into HCC cells, and found the *PKM2* mRNA level was significantly reduced in the miR-122 cells, compared to the controls (Fig. 3E). The endogenous protein level of PKM2 was also decreased upon ectopic expression of miR-122 (Fig. 3F). In accordance with this observation, silencing miR-122 expression by using miR-122 inhibitor upregulated the protein and mRNA levels of PKM2 in HepG2 cells (Fig. 3E-F). In addition, in our pervious mass screening of potential miR-122 targets using luciferase assay, we found the activity of a luciferase construct bearing 3’UTR of *PKM2* was significantly reduced upon expression of miR-122 [6]. Collectively, the data showed that *PKM2* is a direct target of miR-122.

### miR-122 regulates PKM2 mediated metabolism

**Figure 4:**
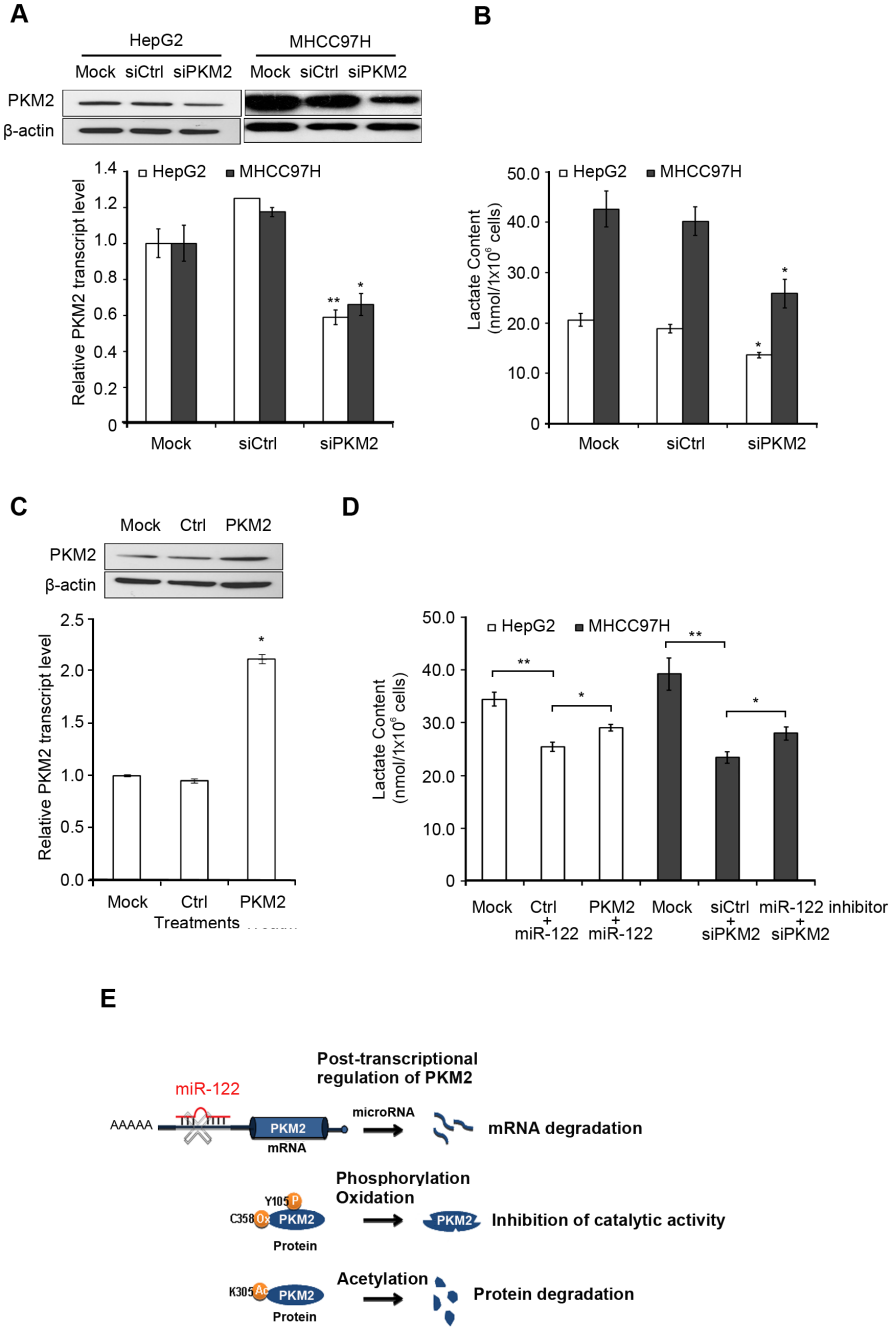
Repression of PKM2 underlies miR-122-dependent phenotypes *in vitro*. **(A)** Protein (upper panel) and mRNA levels (lower panel) of PKM2 in HepG2 or MHCC97H after transfection with siRNA targeted to PKM2 (siPKM2) or with siRNA control (siCtrl). (**B**) Lactate content of cells after siPKM2 or siCtrl transfection. Data were derived from three independent experiments. (**C**) Protein (upper panel) and mRNA levels (lower panel) of *PKM2* after transfection of PKM2 expression plasmid (PKM2). (**D**) The reduction of lactate content upon transfection of miR-122 expression plasmid (miR-122) or siPKM2 was abolished with co-transfection of PKM2 or miR-122 inhibitor, respectively (compared to mock). (**E**) Hypothetical model of miR-122 targeting *PKM2* in HCC metabolisms. *p<0.05, ** *p*<0.01, Error bars represent S.E.M.

To confirm PKM2 is involved in miR-122 regulated metabolic process, we examined whether the reduction of PKM2 lowers lactate production resembling the effect of miR-122 overexpression. We first assessed the suppression efficiency of siPKM2 RNAi duplex. As shown by real-time qPCR data, the siPKM2 gave a 41% reduction on *PKM2* expression (Fig. 4A) that is similar to the effect of miR-122 overexpression in HepG2. Similar results were observed in siPKM2 transfected MHCC97H cells. Next, we transfected the two cell lineswith either siPKM2 or siCtrl, and performed a lactate assay at 48 hours. A significant reduction of lactate production was observed with the siPKM2 but not with the control (Fig. 4B).

On the other hand, PKM2 could counteract the effect of miR-122 on lactate production. HepG2 cells were co-transfected with both the miR-122 and the PKM2 decoy (the plasmid expressed the entire *PKM2* coding region but lacked the 3’UTR, i.e. no miR-122 binding site),and MHCC97H cells were co-transfected with both siPKM2 and miR-122 inhibitor. Our data showed overexpression of PKM2 decoy obviously abrogated the miR-122 suppression effect on lactate production, while silencing endogenous miR-122expression reversed the effect of siPKM2 on lactate production (Fig. 4C and 4D), supporting the miR-122 and PKM2 regulatory relationship on cancer metabolism.

## Discussion

MiR-122 plays an important role in maintenance of normal physiological metabolism in the liver. Dys- or down-regulation of miR-122 is frequently implicated in development of cirrhosis and liver cancer and tumor metastasis [3], [4], [14] as spontaneous liver tumors were observed in Mir122a–/– mice, whereas reduced disease manifestation and tumor incidence when miR-122 expression were restored. In our previous study, we did not found significant correlation of miR-122 with overall survival and disease-free survival rates, although there was a tendency. However, miR-122-targeted genes that are enriched in metabolic pathways significantly associated with HCC survival rates [2]. The connection between PKM2 and miR-122 was observed during differentiation process of human embryonic stem cell (hESCs) into hepatocytes [15]. Furthermore, overexpressing miR-122 leads to hESCs and HCC cells self-renewal and proliferation deficiency [15]. The present study provides new evidences that miR-122 targets *PKM2* metabolic enzyme which plays a pivotal role in aerobic glycolysis having significant association with poor clinical outcomes of the HCC patients. We also demonstrated the strong anti-correlation of miR-122 and *PKM2* expression levels in the cohort of 94 HCC samples. Targeted delivery of miR-122 into HCC may have potential therapeutic values by reversing aerobic glycolysis observed in cancer cells into normal physiologic metabolic pathways via promoting oxidative phosphorylation.

Increased glycolysis has an essential role in supporting HCC development. HCC tumors appeared to have increased expression of glycolytic genes, such as *PKM2*, *hexokinase*, *LDHA*, and *GLUT-1*, and modulation of these genes could affect HCC cell growth [16], [17]. A recent report showed that HCC patients, whose tumor had higher glucose uptake, had shorter survival times. In addition, tumors with elevated Ki-67 labeling index (higher proliferation rate) were accompanied with increased expression of glycolytic genes [18]. Therapeutics targeting HCC metabolism is a relatively unexplored area and should deserve more attention given the evidences these years supporting its essential role in tumor development.

PKM2 promotes aerobic glycolysis and tumorigenesis in many tumor types, such as lung [13], prostate [19], and glioma [20]. The gene is under a complex circuit of regulation: from transcriptional [21] to post-transcriptional levels. Here, we showed *PKM2* transcription is inhibited by miR-122. Other studies showed that phosphorylation [16], acetylation [17], and oxidation [22] at the post-transcriptional level also affect *PKM2* expression and/or function by modulating its enzyme activity or promoting protein degradation (Fig 4E). Nevertheless, it is not clear to us, which one of these regulatory mechanisms plays a central role in controlling *PKM2* contextually and temporally. The level of PKM2 needs to be controlled at a precise level, and either higher or lower level of PKM2 could inhibit tumorigenesis [23]. As such, both the inhibitors and activators of PKM2 have been explored in pre-clinical stage [24], [25]. There are heated discussions on how to target PKM2 in cancer metabolisms, and a deeper understanding of the role of miR-122 in the complex regulatory circuits for the metabolic pathway may lead to new approach in cancer treatment.

In addition to *PKM2*, a cohort of metabolic genes is also under the control of miR-122, and our data analysis revealed that miR-122 is significantly correlated with a battery of glycolytic genes (*PKM2*> *ALDOA*> *GNPDA1* >*PFKFB2* >*AKR1B10*). Furthermore, recent molecular profiling studies have shown genes affected by miR-122 modulation are significantly enriched in metabolic genes [2], [6], suggesting that miR-122 may control several distinct steps of metabolism. Indeed, animal models with modulated miR-122 level revealed alteration in the metabolic phenotypes [3].

In conclusion, the present study demonstrated a regulatory role of miR-122 in HCC metabolism, by targeting *PKM2* (and likely other glycolytic genes) that is involved in aerobic glycolysis supporting cancer cells survival and proliferation. Many of the HCC patients are often, and unfortunately, being excluded from the surgical resection or chemotherapy because of their poor underlying liver functions. Thus it is worth of investigating the due diligence of miR-122 therapeutic functions – by improving the liver function and by inhibiting cancer metabolisms, to benefit our HCC patients.
